# Feasibility and Acceptability of Delivering Pivotal Response Treatment for Autism Spectrum Disorder via Telehealth: Pilot Pre-Post Study

**DOI:** 10.2196/32520

**Published:** 2022-09-06

**Authors:** Krista N Drapalik, David Grodberg, Pamela Ventola

**Affiliations:** 1 Center for Autism and Related Disabilities University at Albany State University of New York Albany, NY United States; 2 Yale Child Study Center Yale University New Haven, CT United States

**Keywords:** autism spectrum disorder, ASD, pivotal response treatment, PRT, telehealth, parent-implemented intervention, parent training, pediatrics, autism, children, digital health, online modules, online health, online treatment, pilot study, communication

## Abstract

**Background:**

Pivotal response treatment (PRT), an evidence-based and parent-delivered intervention, is designed to improve social communication in autistic individuals.

**Objective:**

The aim of this study was to assess the feasibility, acceptability, and clinical effects of an online model of PRT delivered via MindNest Health, a telehealth platform that aims to provide self-directed and engaging online modules, real-time coaching and feedback, and accessible stepped-care to large populations of parents seeking resources for their autistic children.

**Methods:**

Male and female autistic children, aged 2-7 years with single-word to phrase-level speech, and their parents were eligible to participate in the study. Families were randomized to the online parent training condition or control condition. The online component of the intervention consisted of eight 20-minute online courses of content describing parent training principles in PRT. Four 1-hour videoconferences were held after course 1, course 3, course 5, and course 8. Parents were given 1-2 weeks to complete each course. Parents completed the Client Credibility Questionnaire (CCQ) at week 2 and at the study endpoint, as well as the Behavioral Intervention Rating Scale (BIRS) at the study endpoint to assess parental expectancies, and treatment acceptability and effectiveness.

**Results:**

Nine of 14 participants completed the study curriculum in the online parent training condition, and 6 of 12 participants completed the control condition. Thus, a total of 58% (15/26) participants across both groups completed the study curriculum by study closure. Within the online parent training condition, there was a significant increase in mean CCQ total scores, from 25.38 (SD 3.25) at baseline to 27.5 (SD 3.74) at study endpoint (*P*=.04); mean CCQ confidence scores, from 6.0 (SD 1.07) at baseline to 6.75 (SD 0.89) at study endpoint (*P*=.02); and mean CCQ other improvement scores, from 5.25 (SD 0.89) at baseline to 6.25 (SD 1.28) at study endpoint (*P*=.009). Within the control condition, a modest increase in mean CCQ scores was noted (Confidence, difference=+0.25; Recommend, difference=+0.25; Total Score, difference=+0.50), but the differences were not statistically significant (Confidence *P*=.38, Recommend *P*=.36, Total Score *P*=.43). Among the 11 parents who completed the BIRS at the study endpoint, 82% (n=9) endorsed that they *slightly agree* or *agree* with over 93% of the Acceptability factor items on the BIRS.

**Conclusions:**

The feasibility of this online treatment is endorsed by the high rate of online module completion and attendance to videoconferences within the online parent training group. Acceptability of treatment is supported by strong ratings on the CCQ and significant improvements in scores, as well as strong ratings on the BIRS. This study’s small sample size limits the conclusions that can be drawn; however, the PRT MindNest Health platform holds promise to support parents of autistic children who are unable to access traditional, in-person parent-mediated interventions for their child.

## Introduction

### Background

Autism spectrum disorder (ASD) is a neurodevelopmental disorder that is characterized by persistent social communication deficits and the presence of restricted and repetitive behaviors [1]. Currently, it is estimated that 1 in 44 people are diagnosed with ASD within the United States, and males are 4.2 times as likely to hold an ASD diagnosis than females [2]. Early diagnosis and intervention efforts aim to prevent or mitigate the severity of symptoms associated with ASD, and provide autistic children with opportunities for early socialization and communication development [3,4]. Consequently, early intervention has been linked to improved long-term outcomes in autistic children. With this understanding, the development of interventions for young children have emerged and support the importance of targeting the areas of social communication in young autistic children.

Pivotal response treatment (PRT), an evidence-based and parent-delivered naturalistic developmental behavioral intervention (NDBI), is designed to improve social communication in autistic individuals by addressing core deficits in motivation [5]. Research supports the efficacy of PRT utilizing a parent-delivered approach as apparent by increased child eye contact, directed positive affect, social communication, and engagement following the intervention [6,7].

Unfortunately, families often lack access to evidence-based behavioral interventions. It is estimated that an average time lag of 3 years exists between the time of diagnosis to the start of early intervention services for autistic children [8]. Contrary to popular belief, an early diagnosis does not translate to immediate early intervention services, as parents face uncertainty, years-long waitlists, shortages of providers, and competing time demands postdiagnosis [8]. Even when an intervention opportunity becomes available, barriers to obtain such services are extensive, such as distant location, lack of transportation, high costs, limited insurance coverage, parents’ limited time availability, need for childcare, and lack of trained staff [9]. Additionally, parent engagement is limited, and attrition rates are high even among families with access to parent training programs. A recent literature review of 262 behavioral parent training studies found that over 25% of participants who met the inclusion criteria declined to enroll and an additional 26% of participants dropped out during treatment [10]. Moreover, several challenges exist that impede the implementation of parent-mediated interventions that provide feasible, approachable, and acceptable treatment alternatives to parents of autistic children.

To overcome barriers to high-quality care, telehealth—the provision of mental health or medical services via various modes of technology—has recently been implemented to facilitate access to parent-mediated interventions [1]. Telehealth allows for the dissemination of interventions in a variety of formats, ranging from self-directed courses to videoconferencing with a trained clinician.

Research comparing child outcomes of parent-mediated telehealth versus in-person applied behavior analysis interventions reported equitable success in reducing problem behaviors in autistic children, regardless of the modality by which parent coaching was delivered (ie, in person or via remote videoconferencing) [11]. Moreover, data from a pilot randomized controlled trial examining the effect of self-directed versus therapist-assisted parent-mediated telehealth interventions for autistic children found increased social skills only in children randomized to the therapist-assisted group [12]. In support of these findings, studies investigating telehealth adaptations of PRT via self-directed online modules found that following the online modules, parents successfully implemented PRT with fidelity; however, parents reported that a feedback or coaching component would have been increasingly helpful in conjunction with the self-directed media [13,14]. Self-directed parent-mediated interventions with no direct therapist involvement lack valuable opportunities to build a therapeutic alliance and rely heavily upon parent buy-in [14]. This not only illustrates the value of professional support in parent-mediated telehealth interventions but also the importance of utilizing a combined intervention approach that provides self-directed and remote parent-coaching facets in future adaptations. In addition to evaluating child outcomes in parent-mediated telehealth interventions, recent research has considered the feasibility and acceptability of such approaches to delineate if parent-mediated telehealth interventions can become a viable alternative to in-person interventions in the future. There is increasing literature investigating, and consequently supporting, the feasibility of parent-mediated telehealth interventions, wherein several studies reported high parent satisfaction and high treatment retention in addition to positive child outcomes [9,15].

### Study Objectives

The aim of this study was to examine a telehealth NDBI: online PRT via the MindNest Health platform [16]. The purpose of this pilot study was to test the feasibility and acceptability of a novel intervention delivery model for autistic children. Feasibility will assess the plausibility and practicality of online PRT for families of autistic children via parent attendance throughout the intervention. Acceptability will assess if online PRT meets the needs of autistic children and their parents via parental satisfaction, confidence, and perceived treatment success. MindNest takes existing evidence-based skills and strategies used in NDBIs, and delivers them using brief and focused animated simulations in modular pieces. Moreover, MindNest provides active coaching and real-time feedback to parents in addition to self-directed online courses. Finally, MindNest is designed to be integrated into a stepped-care model of care, working to serve large populations of parents who are waiting for services or seeking to supplement existing services they are receiving.

## Methods

### Participants

Participants were recruited via local flyer postings and referrals from specialty clinics in New Haven, Connecticut, and the surrounding areas. Thirty-seven families expressed interest in participating, 11 of whom did not meet the inclusion criteria. A total of 26 autistic children, aged 2-7 years, and their parents were consented and enrolled in this study.

Inclusion criteria were as follows: (1) children aged 2-7 years with single-word to phrase-level speech; (2) a Diagnostic and Statistical Manual of Mental Disorders, Fifth Edition (DSM-5) diagnosis of ASD, based on assessment with the Autism Diagnostic Observation Schedule, Second Edition (ADOS-2) by a licensed provider [1,17]; (3) access to a mobile device and/or computer device with internet; (4) speak English fluently as a primary or secondary language, as endorsed via self-report; and (5) agree not to initiate new mental health treatments for their child for the duration of the study. Participants were excluded if they had received parent training previously, or if the child had a medical or psychiatric condition that required immediate clinical attention. Multiple parents/caregivers per child were welcome to participate in the study. In the event of multiple parents participating, a “primary” caregiver, who was available for all videoconferencing sessions and assessment measures, was determined to maintain continuity.

### Ethics Considerations

The Yale University institutional review board approved this intervention study on October 10, 2018 (reference number: 2000021538). After telephone screening, written informed consent and video consent were obtained from a parent or legal guardian, and verbal assent was obtained from the children at the beginning of the visit.

### Measures

#### Demographic Information

At baseline, parents provided demographic information, including caregiver gender and age, as well as their autistic child’s gender and age.

#### Medical History

At baseline and the study endpoint (week 10), parents completed a survey related to medical history and concomitant treatments (medication or psychosocial intervention) of their child, including the type of treatment, purpose, frequency or dose of treatment, start date, and stop date.

#### Client Credibility Questionnaire

The Client Credibility Questionnaire (CCQ) [18] is a 4-item, parent-rated measure designed to evaluate parental confidence in the efficacy and logistical nature of the treatment. Parents were asked to provide ratings about how logical the parent training seemed, how confident they are that it would be successful, and how confident they would be in recommending the parent training program to a friend. Ratings were made on an 8-point scale, ranging from 0 (not at all logical/confident) to 8 (very logical/confident), with a total score range of 0 to 32. Treatment posed the possibility of inducing different expectancies, and consequently contributing to differences in treatment response. Thus, the CCQ was used to assess parent expectancies [18]. Parents completed the CCQ at the end of week 2 and again at the study endpoint (week 10). This measure was completed at week 2 because, given the nature of the assessment measure, parents needed initial exposure to the treatment program before providing a rating.

#### Behavioral Intervention Rating Scale

The Behavioral Intervention Rating Scale (BIRS) is a 24-item, parent-rated measure designed to evaluate treatment acceptability and efficacy via three factors: Acceptability, Effectiveness, and Time of Effect [19]. Parents responded using a 5-point scale, ranging from 1 (strongly disagree) to 5 (agree), with a total score range of 24 to 120. BIRS Acceptability factor items (items 1-15) are used to indicate if an intervention can be deemed acceptable based on the number of *slightly agree* and/or *agree* responses from participants. Parents completed the BIRS only at the study endpoint (week 10).

### Design

Families were randomized to the online parent training condition or to the control condition, with children matched according to sex and age. Parents randomized to the online parent training condition received access to a 10-week consultative parent-training model in PRT via the MindNest Health online platform. In the control condition, parents were provided with a written copy of the PRT manual (“PRT Pocket Guide”) to read independently [5]. All parents were instructed to practice PRT for 1 hour per day a minimum of 5 days per week, and were asked to submit a log indicating the dates and duration of their practice prior to each videoconferencing session. Specific days to practice were not prescribed; however, based on the clinical experience of the authors, 5 days per week was deemed to be a reasonable and feasible request for an at-home, independent practice of PRT. Additionally, both groups completed two follow-up videoconferencing sessions with a clinician in which parents were asked to perform PRT with their child for 10 minutes. These sessions took place in week 12 and week 14 and were recorded. Following successful completion of the control condition and all study assessments, families who were randomized into the control condition were provided access to MindNest Health and given the opportunity to receive videoconferencing with a PRT clinician.

### MindNest Health

MindNest Health is an online telehealth platform that delivers evidence-based and scalable education, training, and support for parents who have a child with mental health or behavioral problems [16]. Within this study, MindNest Health was the telehealth platform used to deliver PRT to families randomized to the online parent training condition. The company was developed by a group of Yale University faculty and students.

**Figure 1:**
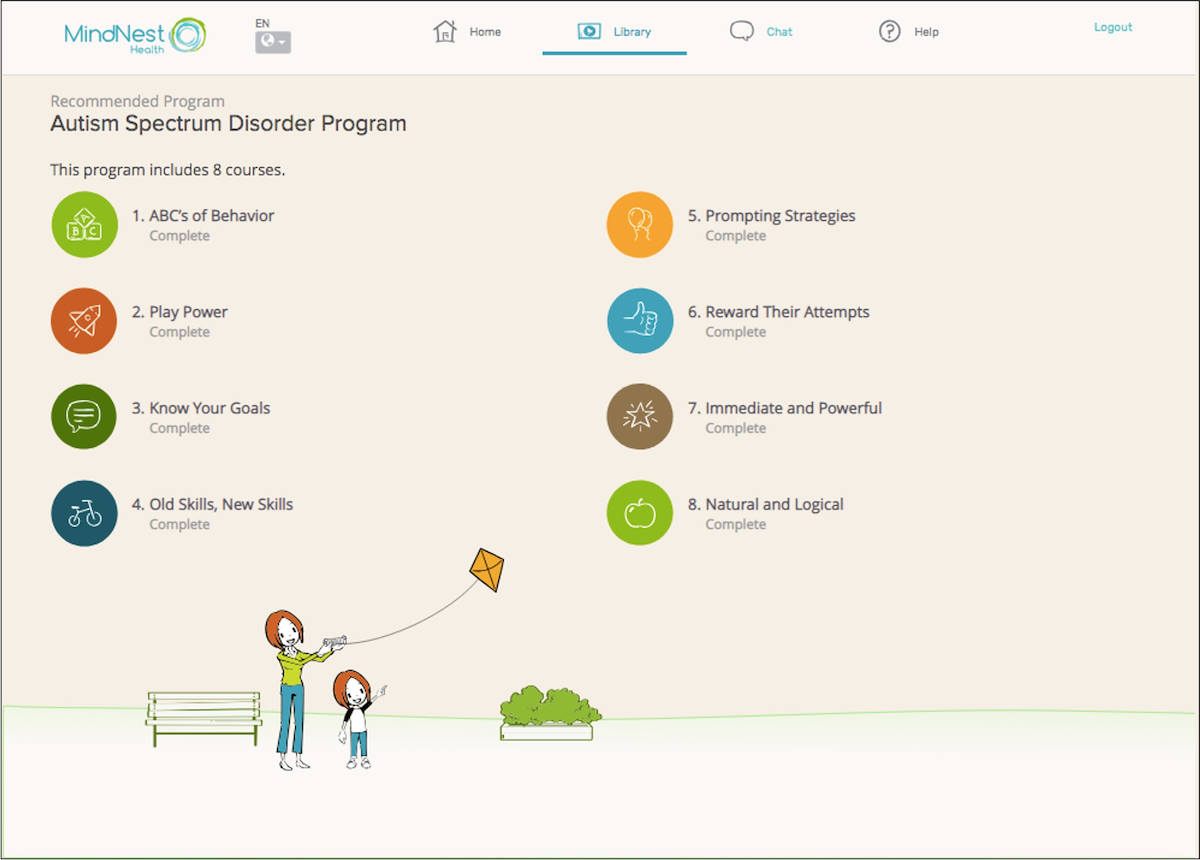
MindNest Health interface displaying the eight offered modules.

**Figure 2:**
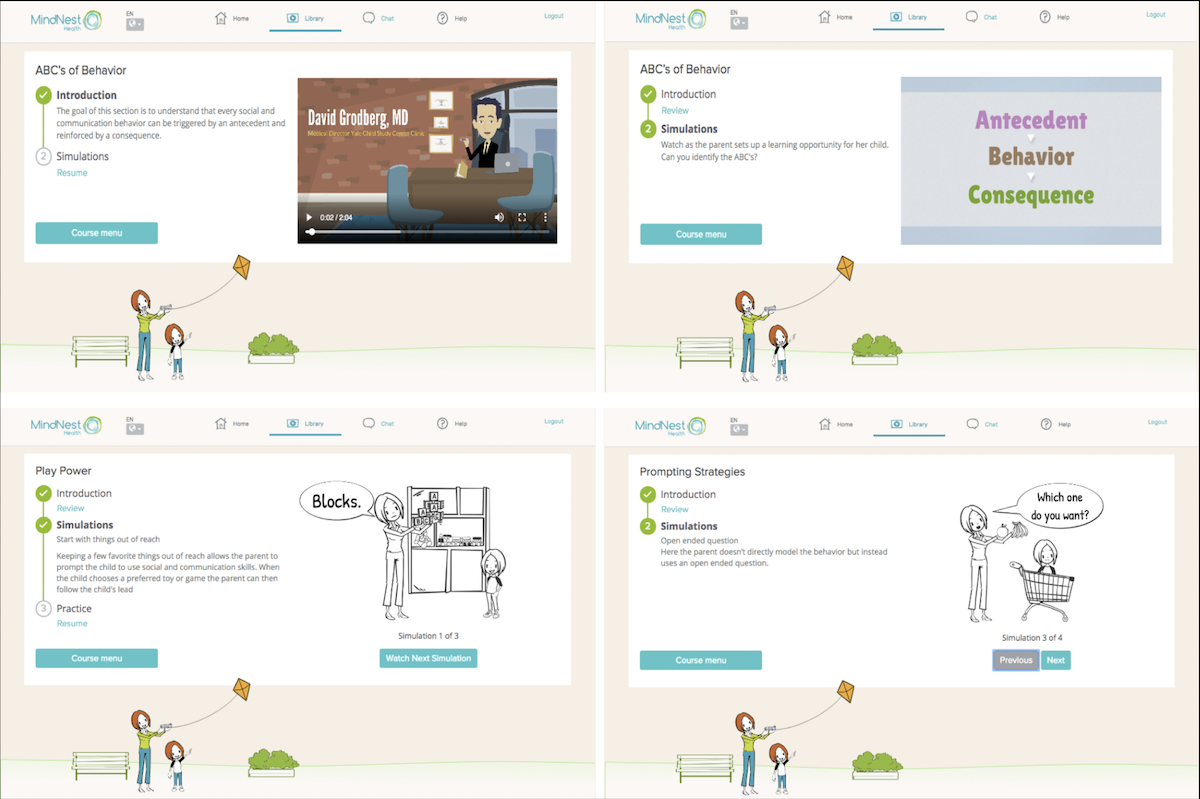
MindNest Health courses, including didactic information as well as demonstrations of behavioral strategies using animated simulations. For example, simulations demonstrate how a parent can prompt a child to ask or respond to questions across different settings.

The MindNest Health offering includes an integrated learning management system that delivers parent education and parent training in behavioral interventions (Figure 1). The lessons incorporate a blend of didactic information as well as demonstrations of PRT principles via short, animated simulations of parent-child dyads (Figure 2). Parents were able to move through animations at their own pace and are given opportunities to practice the skills and strategies as they move through the lesson (Figure 2). The platform has undergone professional user testing with parents of autistic children and children with other developmental delays, and the feasibility and acceptability of the platform were supported in a recent study [9]. The content was delivered in parent-friendly and accessible language, and the PRT principles are illustrated using animated simulations of a parent and child with voiceover from a narrator. Video examples of the PRT principles are a critical component to teaching the approach, and the technological advances in instructional design allowed for the creation of exact models illustrating complex constructs.

### Intervention

After the initial telephone screening study visit, parents in the online parent training condition were given instructions to access the MindNest Health platform. The online component of the intervention consisted of eight 20-minute online courses of content describing parent training principles in PRT (Figure 1). Parents completed each course at their own pace and were asked to complete one course per week for the first 8 weeks of the intervention. All parents completed the following courses chronologically: “The ABCs of Behavior”; “Play Power”; “Know Your Goals”; “Old Skills, New Skills”; “Prompting Strategies”; “Reward Their Attempts”; “Immediate and Powerful”; and “Natural and Logical.” These courses respectively taught parents an overview in behavioral principles, an overview of PRT, how to create contingencies for behavior, the importance of maintenance and acquisition task, how to use behavioral prompting to support behavior, and how to reinforce behavior. Table 1 provides the sequence and description of intervention topics.

Four 1-hour videoconferences were held after course 1, course 3, course 5, and course 8. Parents were given 1-2 weeks to complete each course. These intervals were selected to spread the videoconference sessions throughout the study duration, but also specifically following core and conceptually distinct courses (eg, overview of behavioral strategies in general, overview of PRT and the importance of behavioral contingencies for beginning PRT, use of prompting, reinforcing behavior). These videoconferencing sessions took place using either FaceTime or Vidyo, a Yale-owned videoconferencing program that did not require a software download. Participants had the option to videoconference on a tablet, smartphone, or computer. Videoconferencing sessions were recorded. Videoconferences were conducted by a trained PRT clinician, and gave families an opportunity to apply, implement, and utilize the principles of PRT that the parents were learning online. The trained clinician provided feedback to parents about their individual implementation of PRT, in accordance with a practice-with-feedback model [20]. The lead clinician is a licensed psychologist with extensive experience working with autistic children and implementing PRT. The other PRT clinicians included three female, bachelor-level staff who spoke English as their primary or secondary language and had 1-5 years of experience working with autistic children. Prior to study enrollment, all study clinicians underwent intensive training in PRT and each clinician was required to meet fidelity for PRT implementation prior to participant contact. The lead clinician provided supervision and feedback to PRT clinicians throughout the duration of the study.

**Table 1:** Pivotal response treatment (PRT) online courses and videoconferencing session timeline.

Timeline		Course	Topic
Week 1		ABCs of Behavior	Overview in behavioral principles
**Videoconferencing session 1**			
	Week 2	Play Power	Overview of PRT, creating learning opportunities during play interactions
	Week 3	Know Your Goals	How to create contingencies for behavior and attainable target goals
**Videoconferencing session 2**			
	Week 4	Old Skills, New Skills	Importance of using both maintenance and acquisition tasks to increase motivation
	Week 5	Prompting Strategies	How to use behavioral prompting to support behavior
**Videoconferencing session 3**			
	Week 6	Reward Their Attempts	Reinforcing attempts, even when the target goal is not achieved
	Week 7	Immediate and Powerful	Reinforcing behavior via highly motivating rewards that are given immediately following behavior
	Week 8	Natural and Logical	Reinforcing behavior via rewards that are natural and logically connected to the child’s actions
Videoconferencing session 4			

## Results

### Participants

Fourteen parent-child dyads were randomized to the online parent training condition. Participants included children, 9 males (mean age 4.56 years) and 5 females (mean age 4.24 years), and their parents, 9 mothers (mean age 39.77 years) and 5 fathers (mean age 43.33 years). Twelve parent-child dyads were randomized to the control condition. Participants included children, 8 males (mean age 4.65 years) and 4 females (mean age 4.71 years), and their parents, 7 mothers (mean age 41.07 years) and 2 fathers (mean age 40.54 years). Each child is represented by one parent.

### Feasibility and Acceptability

Nine of 14 participants (5 males, 4 females) completed the study curriculum in the online parent training condition, and 6 of 12 participants (5 males, 1 female) completed the control condition. A total of 15 of 26 participants (58%) completed the study curriculum by study closure. Of those who completed the study, 13 of 15 (97%) participants completed all four videoconferences and 100% of participants in the online parent training group completed all four videoconferences. Additionally, 14 of 15 (93%) participants completed at least one endpoint visit after the conclusion of treatment. One participant who completed the intervention did not attend an endpoint visit and three participants failed to return endpoint measures via mail. Of those who withdrew from the study, 6 of 11 (55%) were randomized to the control condition and 3 of 11 (27%) cited time commitment as the reason for withdrawing. Before withdrawing from the study, 8 of 11 (73%) participants completed one or more videoconferences and received treatment materials (ie, treatment manual or access to MindNest Health online courses).

### Parental Perception and Acceptability of Treatment

#### CCQ Outcomes

Of the participants who completed the intervention and attended an endpoint visit, 12 of 15 parents (n=8, online parent training condition; n=4, control condition) completed and returned the CCQ before and after treatment. At both the baseline and endpoint, parents in the online parent training condition endorsed higher scores on the CCQ in comparison to those of parents in the control condition (baseline mean difference=+3.13, endpoint mean difference=+4.75); however, no significant differences emerged between group means (*P*=.32). The mean CCQ total score at baseline was 25.38 (SD 3.25) and was 27.5 (SD 3.74) at the endpoint for the online parent training condition. Within the control condition, the mean CCQ total score was 22.25 (SD 7.46) at baseline and was 22.75 (SD 6.18) at the endpoint.

There was a significant increase in mean CCQ total scores from 25.38 (SD 3.25) at baseline to 27.5 (SD 3.74) at the study endpoint (*P*=.04) among parents who completed the online parent training condition. Additionally, parental confidence in the intervention’s ability to reduce their child’s symptoms increased significantly from 6.0 (SD 1.07) at baseline to 6.75 (SD 0.89) at the study endpoint (*P*=.02). Among the same group, a significant increase was found in mean scores, indicating perceived success of the treatment’s ability to improve nontargeted areas such as sadness, anxiety, and schoolwork, from 5.25 (SD 0.89) at baseline to 6.25 (SD 1.28) at the study endpoint (*P*=.009). While modest increases endured in parental confidence of treatment success (mean difference=+0.25), likelihood of recommending the treatment to others (mean difference=+0.25), and CCQ total score (mean difference=+0.50) within the control group from baseline to the endpoint, no significant differences were found (Confidence *P*=.38, Recommend *P*=.36, Total Score *P*=.43).

#### BIRS Outcomes

There were 11 parents (n=7 in the online parent training condition and n=4 in the control condition) that completed the BIRS at the study endpoint after completing treatment. Within the online parent training group, 6 of 7 parents certified that they *slightly agree* or *agree* with the intervention’s ability to improve their child’s difficulties on 14 of the 15 BIRS Acceptability factor items (>93%). All parents in the control condition responded they *slightly agree* or *agree* with the efficacy of the intervention on 13 of the 15 BIRS Acceptability factor items (>87%). Combined, 9 of 11 (82%) parents endorsed they *slightly agree* or *agree* with over 93% of the Acceptability factor items on the BIRS (14 of 15 items). Moreover, 8 of 15 (53%) Acceptability factor items were rated *slightly agree* or *agree* by all parents. Additionally, the highest rating (5, *agree*) was indicated by 73% (8 of 11) parents in their willingness to use the intervention in a home setting and agreement that the intervention would *not* result in negative side effects for their child (Table 2).

**Table 2:** Behavior Intervention Rating Scale (BIRS) Acceptability factor item data.

BIRS items^a^	Online parent training (n=7), mean (SD)	Control (n=4), mean (SD)
Item 1. This would be an acceptable intervention for my child’s problem behavior	4.57 (0.54)	4.75 (0.50)
Item 2. Most parents would find this intervention appropriate for behavior problems in addition to the one described	4.29 (0.76)	4.25 (0.96)
Item 3. The intervention should prove effective in changing my child’s problem behavior	4.29 (0.49)	4.25 (0.50)
Item 4. I would suggest the use of this intervention to other parents	4.57 (0.54)	4.25 (0.50)
Item 5. The child’s behavior problem is severe enough to warrant use of this intervention	4.0 (1.0)	4.5 (1.0)
Item 6. Most parents would find this intervention suitable for the behavior problem described	4 (0.82)	4.25 (0.50)
Item 7. I would be willing to use this in the home setting	4.71 (0.49)	4.75 (0.50)
Item 8. The intervention would *not* result in negative side effects for my child	4.57 (0.54)	5.0 (0.00)
Item 9. The intervention would be appropriate for a variety of children	3.86 (0.38)	4.5 (0.58)
Item 10. The intervention is consistent with those I have used in the home setting	4.29 (0.49)	4.0 (0.82)
Item 11. The intervention was a fair way to handle my child’s problem behavior	4.43 (0.79)	4.5 (0.58)
Item 12. The intervention is reasonable for the behavior problem described	4.43 (0.54)	4.5 (0.58)
Item 13. I like the procedures used in the intervention	4.57 (0.54)	4.25 (0.96)
Item 14. This intervention was a good way to handle my child’s behavior problem	4.57 (0.54)	4.5 (0.58)
Item 15. Overall, the intervention would be beneficial for my child	4.67 (0.52)	4.5 (0.58)

^a^Items are scored on a scale of 1-5; higher scores indicate higher agreement.

## Discussion

### Principal Results

This study examined the feasibility and acceptability of an individualized parent-mediated treatment for autistic children. The feasibility of this online treatment is endorsed by the high rate of online module completion and attendance to videoconferences (>70%) within the online parent training group. The dropout rate within the parent training condition was relatively low (36%) and was higher within the control condition (50%). Three families cited time commitment as the reason for withdrawal; however, all three families were experiencing outside familial stressors unrelated to treatment they had to address. The other reasons for dropout are unclear; however, in all cases, parents appeared to have difficulty responding to the phone calls and emails of the study clinicians, particularly following a delay (ie, post treatment follow-up) or when consistent communication was not established (ie, control condition).

Strong ratings on the CCQ and significant improvements in scores suggest that the treatment was highly acceptable to participating parents within the online parent training condition. Parents endorsed significantly increased feelings of confidence in the intervention’s ability to reduce their child’s symptoms and improve nontargeted areas such as sadness, anxiety, and schoolwork. Within the control condition, parents did not endorse scores of the same magnitude, and reported lower ratings on the CCQ at both baseline and the study endpoint. This indicates that parents in the online parent training condition found the intervention to be more acceptable than parents who did not participate in the online courses via MindNest Health and only received the PRT manual.

Further, strong ratings on the BIRS support the high acceptability of the intervention to participating parents. A majority of parents (>80%) agreed with all but one of the Acceptability factor items on the BIRS. General themes of parents’ ratings included willingness to use the intervention in a home setting and agreement that the intervention would *not* result in negative side effects for their child. Moreover, parents in both conditions expressed satisfaction in the treatment.

The PRT MindNest Health platform holds promise to support parents of autistic children who are unable to access traditional, in-person parent-mediated interventions for their child. As this is a pilot, open feasibility study, we cannot confirm, and did not presume, that the change in clinical symptoms was due to MindNest Health rather than parents’ expectations or attention from study personnel.

From an assessment point of view, the combination of a platform comprising general content with videoconferences to individualize the skills garners much value. The online videoconferences provide opportunity for a “naturalistic” observation in ways that are otherwise missed in a clinical setting. For instance, during videoconferences, the disruptions of neighbors or siblings and the logistical challenges of the house setup became more apparent. Further, a brief online intervention serves as a thorough, low-cost assessment of patients’ and families’ needs, strengths, and vulnerabilities, gauging whether parents and patients would be able to engage in routine therapy.

### Limitations

This study’s small sample size and limited parent demographic information limits the conclusions that can be drawn regarding parents’ ratings of acceptability. A future iteration of this study should include additional efficacy measures and demographic information (ie, marital status, racial and ethnic identity, education) to determine if children show improvements in their social communication and behavior skills following the treatment and to understand sociodemographic factors that may influence the feasibility and acceptability of this telehealth intervention. Further, while escalated rates of attrition are evident in similar online parent training programs, the high attrition rate, particularly in the control group, may impact the generalizability of the results and should be considered when developing future iterations of this study [10,12,14,21].

Another important limitation of this study is the requirement for parents to have their own technological devices and internet access. While an aim of this study was to increase accessibility to early evidence-based interventions, this requirement precluded families who did not have access to the aforementioned technologies from enrolling. A requirement such as this may have limited study enrollment to only families of higher socioeconomic status (SES). It is acknowledged that telehealth may not be a viable solution to families of low SES due an inaccessibility of required technologies. Future studies can aim to address these issues by providing devices to families or incorporating an in-person alternative.

### Conclusions

The PRT MindNest Health platform is a promising intervention that has the potential to increase access to evidence-based interventions for parents of autistic children who are unable to access in-person services via engaging online modules and videoconference coaching. Online treatments hold great potential in their ability to improve access to care. Thus, future studies are required to determine the efficacy of parent-mediated telehealth interventions and the possibility of such interventions serving as a viable and effective alternative to in-person evidence-based interventions.

## Abbreviations

ADOS-2: Autism Diagnostic Observation Schedule, Second Edition
ASD: autism spectrum disorder
BIRS: Behavioral Intervention Rating Scale
CCQ: Client Expectancies Questionnaire
DSM-5: Diagnostic and Statistical Manual of Mental Disorders, Fifth Edition
NDBI: Naturalistic Developmental Behavioral Intervention
PRT: Pivotal Response Treatment
SES: socioeconomic status
